# 

**Published:** 1919-02-01

**Authors:** 


### THE COLLEGE OF NURSING (Limited by Guarantee).

### LONDON CENTRE

(Hon. Secretary, MISS BIGGAR, St. Thomas's Hospital, S.E. 1.)

A MEMBERS' meeting will be held on Tuesday, February 4, at 7.30 P.M., at the College of Ambulance, No. 3 Vere Street, W., when Dr. Jane Walker will deliver an address on Public Health.

### GLASGOW CENTRE.

(Hon. Sec., Miss LINDSAY, Fever Hospital, Belvedere.)

THERE was a crowded attendance at the meeting of this Centre on January 21. Miss Melrose, R.R.C., was in the chair, and on the platform were Miss Gregory Smith, R.R.C., Miss Merchant, and Miss Lindsay, Hon. Secretary. Professor Glaister gave a most lucid and interesting exposition of the two Bills which are being put forward for State Registration of Nurses. He contrasted them clause for clause, pointing out the democratic character of the College of Nursing Bill. The lecture was listened to with great interest by all present, and many questions were asked and answered. Twenty-eight new members were added to the Centre.

### BIRMINGHAM CENTRE.

(Hon. Sec., Miss COLBURN, 9 Mossley Road.)

THE next meeting will be held at the General Hospital Lecture Theatre, on Monday, February 10, at 6 P.M., when Major Naughton Dunn, R.A.M.C., will lecture on "Modern Orthopædic Surgery." All nurses are cordially invited. Fee 1s. each lecture to non-members.

### GIFTS AND BEQUESTS.

Mr. C. J. Kohler, of Bexhill, left £500 to Hastings Hospital.

Mr. John Locke, of Lee, and late of Barbados, left £1,000 each to the Middlesex Hospital and the London Hospital.

Dr. M. P. James, late lecturer on *materia medica* at the London Hospital and physician to Golden Square Throat Hospital, left £100 to the London Hospital.

Mr. James Lazenby, of Skipton, left £500 to the Royal Infirmary, Bradford, and £250 each to the Blind Institute, Bradford, and the District Nursing Association, Bradford.

The Northampton General Hospital has received £3,000 from Mr. E. T. Hawkins towards a pathological laboratory. Hitherto specimens have been sent to London, and the nearest laboratory has been at Leicester.

Under the will of the late Professor Hume Brown, of Edinburgh University, the sum of £1,500 to found a prize is left to the University, and the residue of the estate is to be divided equally between the Edinburgh Royal Infirmary and the Royal Edinburgh Hospital for Sick Children.

Mrs. Jessie Mills Smith, of Pollokshields, left £10,000 to the Glasgow Royal Infirmary, to endow a ward in remembrance of her late husband; £5,000 each to the Western Infirmary, Glasgow, and the Victoria Infirmary, Glasgow; and £15,000 to other Scottish charities.

Mr. W. S. Carr, of Arthog, Merioneth, left estate valued at £45,122. Subject to family bequests the estate is to be divided between St. Dunstan's Hostel for Blinded Soldiers and Queen Mary's Auxiliary Convalescent Hospitals for the endowment of a permanent hospital for limbless sailors and soldiers.

Mrs. Talbot, of Margam, the gross value of whose estate will be sworn at a figure not exceeding £2,000,000, left £1,000 each to King Edward VII.'s Hospital, Cardiff, and the Swansea General and Eye Hospital, and £300 to the Gower Nursing Association of Queen Victoria's Institute for Nurses.

The late Mrs. Frances Stanborough has bequeathed £100 to the Chelsea Hospital for Women.

The Rev. Richard Herbert Snape, of Eastbourne, has left £1,000 to the Princess Alice Memorial Hospital.

The Corporation of the City of London has made a grant of £50 to the Royal Dental Hospital, Leicester Square.

Mr. W. B. Morris left the residue of his property to be divided between the Children's Hospital, Paddington Green, and St. Mary's Hospital.

London Homœopathic Hospital has received a £1,000 War Bond from Mr. John Mews, to endow "The Capt. John Keith Mews Bed" in the King Edward VII. Ward.

Colonel Arthur Mesham, formerly High Sheriff for Denbighshire and Flintshire, left £100 to the North Denbighshire Infirmary, and £100 to the Alexandra Hospital for Children, Rhyl.

Mr. J. B. Irwin, of Dublin, left the residue of his property, valued at about £3,000, to the British Red Cross Society, in memory of his grandson, Viscount Glentworth, flight commander, killed in the war.

The London Homœopathic Hospital, Great Ormond Street, W.C. 1, has received £1,000 from the Residuary Estate of the late Sir John Howard to endow "The John Howard Bed" in the King Edward VII. Ward.

Mr. William Grantham, of Manchester, left £500 to the Salford Royal Hospital, £100 to the Manchester Royal Infirmary, £1,000 War Loan to Ancoats Hospital for the endowment of a "Sarah Grantham" bed, £500 to the Northern Counties Hospital for Incurables, Mauldeth, and £100 to the Manchester Hospital for Consumption.

Sir Ernest Schiff's will provides a sum of £100,000 to the Charity Commissioners, the income to be applied for pensions for poor persons who have attained fifty-two years of age, and from accident, ill-health, or infirmity are wholly or in part unable to maintain themselves. Amongst specific legacies under the will are £400 each to the Royal Hospital for Incurables and the British Home and Hospital for Incurables.

### Matrons' and Sisters' Appointments.

BERMONDSEY INFIRMARY, ROTHERHITHE.—Miss May Matilda Fivash has been appointed ward sister. Trained at the above-named institution, she has served in Queen Alexandra's Imperial Military Nursing Service Reserve, and has been sister-in-charge of an Auxiliary Military Hospital under the Joint War Committee. Miss Fivash has also done private nursing.

CAMERON HOSPITAL, WEST HARTLEPOOL.—Miss Henrietta Wilson has been appointed night sister. She was trained at the Metropolitan Hospital, London, and has been sister at the Red Cross Hospital, Newton Don, Kelso, N.B.

NOTTINGHAM, CHILDREN'S HOSPITAL.—Miss Ida Berry has been appointed night sister. She was trained at Oldham Royal Infirmary, and has been sister at Birkenhead Children's Hospital, of the military wards at Wigan Royal Infirmary, and at the Victoria Hospital, Blackpool. Miss Berry is a member of the College of Nursing, and has recently been mentioned in despatches for valuable nursing services rendered in connection with the war.

QUEEN VICTORIA COTTAGE HOSPITAL, QUARRY HILL, TONBRIDGE.—Miss Grace Farquhar has been appointed matron. She was trained at St. Bartholomew's Hospital, London, has been night sister at the Bromley Cottage Hospital, night and ward sister and assistant matron and home sister at St. Bartholomew's Hospital, Rochester.

GENERAL INFIRMARY, BURTON-ON-TRENT.—Miss Margaret Williams has been appointed sister. She was trained at the Royal Infirmary, Liverpool.

CITY HOSPITAL NORTH, LIVERPOOL.—Miss Mabel Robinson has been appointed night superintendent. She was trained at the Infirmary, Toxteth, Liverpool, where she has since been sister, and has also been sister at the City Hospital, Grafton Street, Liverpool. Miss Robinson has also done military nursing at Aylesbury.

CITY HOSPITAL EAST, LIVERPOOL.—Miss Rose M. Eddie has been appointed night superintendent. She was trained at Bolton Infirmary and Dispensary and at the Isolation Hospital, Derby. She has been sister at the City Hospital, Grafton Street, Liverpool.

ROYAL HAMPSHIRE COUNTY HOSPITAL, WINCHESTER.—Miss D. Target Fry has been appointed night sister. She was trained at the Royal Infirmary, Leicester, and has been ward sister at the Royal Hampshire County Hospital, besides holding military posts in France and England from August 1914 to the present month.

BOLINGBROKE HOSPITAL, WANDSWORTH COMMON.—Miss Marion Nuttall has been appointed matron. Trained at Manchester Children's Hospital and St. Bartholomew's, she has been sister at the last-named institution and matron of the Royal Victoria Hospital, Dover, during the war period.

### The Book Market.

LIST OF NEW BOOKS RECEIVED FROM THE FOLLOWING PUBLISHERS:—

Henry Frowde, Hodder and Stoughton: "The Hearts of Men." By R. M. Wilson. 6s. net.

John Lane: "The Love of an Unknown Soldier." 3s. 6d. net. "Rhymes of the Red Triangle." Pictures by Joyce Dennys, verses by Hampden Gordon. 4s. 6d. net.

Wm. Heinemann: "War Nursing: What Every Woman Should Know." By Charles Richet. Translated by Helen De Vere Beauclerk. 3s. 6d. net. "Natural Science and the Classical System in Education." By Sir E. Ray Lankester, K.C.B., F.R.S.

Cambridge University Press: "War Neuroses." By John T. MacCurdy. 7s. 6d. net.

Methuen and Co., Ltd.: "Present-Day Applications of Psychology." By Charles S. Myers, M.A., M.D. 1s. net.

John Murray: "A Junior Course of Practical Zoology." By the late A. Milnes Marshall, M.D., and the late C. Herbert Hurst, Ph.D. Eighth Edition. Revised by F. W. Gamble, D.Sc., F.R.S. 12s. net.

Skeffington and Son, Ltd.: "The Prisoner of War in Germany." By Daniel J. McCarthy, A.B., M.D. 12s. 6d. net.

Longmans, Green and Co.: "Twilight Sleep (Scopolamine-morphine Narcosis)." Being the Report of a Special Committee of the Section of Obstetrics and Gynæcology of the Royal Society of Medicine, with a Discussion thereon. 3s. net.

Cassell and Co., Ltd.: "A Manual of Chemistry." By Arthur P. Luff, M.D., and Hugh C. H. Candy, B.A. 12s. net. "Materia Medica and Therapeutics." By J. Mitchell Bruce, M.A., etc., and Walter J. Delling, M.B., etc. Eleventh Edition. 9s. net.

The Scientific Press, Ltd.: "Burdett's Hospitals and Charities, 1918." Edited by Sir Henry Burdett, K.C.B. 12s. 6d. net.

Hodder and Stoughton: "The Life of Dr. Elsie Inglis." By Lady Frances Balfour. 6s. net.

Sampson Low, Marston and Co., Ltd.: "Low's Handbook to the Charities of London." 1s. 6d. net.

Butterworth and Co., Calcutta: "Kala-Azar: Its Diagnosis and Treatment." By Ernest Muir, M.D. R.2 net.

Proprietors of *Country Life*: "The Doings of Donovan." Sketches by J. H. Dowd, with a Foreword by W. Pett Ridge. 3s. 6d. net.

Committee of Organisation: "Transactions of the Sixth International Dental Congress." London. 1914. 30s. net.

J. and A. Churchill: "Sanitation in War." By Lt.-Col. S. S. Lelean. 7s. 6d. net.

Skeffington and Sons, Ltd.: "Humour in Tragedy: Hospital Life Behind Three Fronts." By Constance Bruce.

Macmillan and Co.: "A Health Reader for Girls." By Agnes L. Stenhouse and E. Stenhouse. 3s. net.

John Murray: "Handbook of Physiology." By W. D. Halliburton, M.D. Fourteenth edition. 16s. net.

J. B. Lippencott Co.: "The Koehler Method of Physical Drill." By Capt. Wm. H. Wilbur. 4s. 6d. net. "Hygiene of the Eye." By Wm. Campbell Posey, A.B., M.D. 18s. net.

### Forthcoming Events.

FOUR lectures on Malaria will be delivered at noon on January 31, February 7, 14, and 21, 1919, in the Medical School at King's College Hospital, Denmark Hill, S.E., by Colonel Sir Ronald Ross, consultant in malaria at the War Office and physician for tropical diseases, King's College Hospital. Officers and men of the R.A.M.C. will be welcome. Microscope specimens and lantern slides will be shown at the third and fourth lectures.

